# Resveratrol Attenuates the Proliferation of Prostatic Stromal Cells in Benign Prostatic Hyperplasia by Regulating Cell Cycle Progression, Apoptosis, Signaling Pathways, BPH Markers, and NF-κB Activity

**DOI:** 10.3390/ijms22115969

**Published:** 2021-05-31

**Authors:** Jowon Jang, Junhui Song, Jiyun Lee, Sung-Kwon Moon, Bokyung Moon

**Affiliations:** Department of Food and Nutrition, Chung-Ang University, Anseong 17546, Korea; jjw1832@gmail.com (J.J.); goodabc123@naver.com (J.S.); zzia98@naver.com (J.L.)

**Keywords:** resveratrol, benign prostatic hyperplasia, WPMY-1 cells, proliferation, cell cycle, nuclear factor-κB

## Abstract

Resveratrol can inhibit cell proliferation and metastasis and induce apoptosis. However, the mechanisms of action through which resveratrol inhibits the abnormal proliferation of prostate stromal cells, causing prostatic hyperplasia, have not been fully elucidated. Here, we evaluated the inhibitory effects of resveratrol on cell proliferation associated with prostatic hyperplasia using WPMY-1 cells. Our results showed that resveratrol inhibited the proliferation of WPMY-1 cells via the induction of G_0_/G_1_-phase cell cycle arrest, which was caused by downregulated expression of cyclins and cyclin-dependent kinases regulated by increased p21WAF1 and p27KIP1 expression level. In addition, resveratrol treatment suppressed the phosphorylation of phosphatidylinositol 3-kinase/AKT and extracellular signal-regulated kinase 1/2. The expression levels of molecular markers affecting prostate development were also reduced by treatment with resveratrol. Finally, resveratrol attenuated the binding activity of the transcription factor nuclear factor-κB in WPMY-1 cells, and accelerated apoptotic cell death via intrinsic cascade pathway. These results indicate that resveratrol may be useful for the prevention or treatment of prostatic hyperplasia.

## 1. Introduction

Resveratrol (3,4”,5-trichlorostylbene) is a natural phytoalexin known to have antioxidant, anti-inflammatory, neuroprotective, and immunosuppressive properties, and is found mainly in red wine, berries, and peanuts [1]. Resveratrol has also been shown to prevent cancer and act as an antioxidant and antimutagen in mice in vivo [2]. Previous studies have reported that resveratrol can also be used as an anticancer agent that can inhibit cell proliferation and metastasis, induce apoptosis, and contribute to chemotherapy [3].

Benign prostate hypertrophy (BPH) is a pathological disease associated with aging that occurs in about 50% of men between the ages of 40 and 50 worldwide. Its prevalence continues to increase with age [4,5], and the progression of BPH is triggered by the proliferation of epithelial and stromal cells of the prostate; this results in lower urinary tract symptoms, including painful urination, weak stream, urinary incontinence, and nocturia [6]. Androgenic steroids are required for the embryonic development and pubertal growth of the prostate; studies have assessed the relationship of BPH onset with male hormones, their metabolites, and abnormal prostate growth. From these studies, researchers concluded that changes in steroid levels with aging are associated with cell growth and proliferation in the prostate, resulting in the enlargement of the prostate gland [7,8,9]. Human androgens that play essential roles in the progression of BPH include testosterone and dihydrotestosterone (DHT) [10]. The 5α-reductase enzymes catalyze the synthesis of the active androgen DHT from testosterone. DHT has a high affinity for androgen receptors (ARs), which mediate cell proliferation and promote the differentiation of prostate cells [11]. In addition, prostate stromal cells overexpress autocrine growth factors such as fibroblast growth factors (FGFs) in BPH, thereby promoting cell growth [12]. Moreover, the ratio of apoptosis to proliferation in epithelial and stromal cells is lower in BPH than in normal tissues. Therefore, it is important to modulate normal cell death processes. The decrease in the anti-apoptotic factor Bcl-2 and the increase in the apoptosis factor Bax observed in BPH cells may be due to the decrease in BPH [9,13]. Thus, the regulation of DHT androgens is essential to inhibit BPH progression; this can be achieved by inhibiting 5α-reductases and blocking AR signals. Additionally, cell proliferation could be modulated by controlling the expression of FGF, Bcl-2, and Bax proteins [10,14,15].

All cells, including prostate stromal cells and epithelial cells, regulate division and duplication throughout the cell cycle [16]. The cell cycle can be divided into four stages: the gap before DNA replication (G_1_), the DNA synthesis phase (S), the gap after DNA replication interval (G_2_), and cell division (M) [17]. The G_1_ phase involves the main signal pathways that control cell cycle progression. The cell cycle of mammalian cells is assembled and activated by different cyclin/cyclin-dependent kinase (CDK) proteins, which are expressed and activated at specific points in the cell cycle. The first cyclin/CDK holoenzyme consists of either CDK4 or CDK6, depending on the cyclin D and cell type. Cyclin E and CDK2, which are expressed during G_1_ phase in other mammals, are synthesized later than D-type cyclins, and reach peak expression during late G_1_ phase. Cyclins D and E control the G_1_ phase of the cell cycle, indicating that they can play an essential role in the mammalian G_1_ phase. In addition, cell cycle regulatory complexes can be inhibited by p21_WAF1_ and p27_KIP1_, which are negative regulators of G_1_ phase progression [18,19].

The mitogen-activated protein kinase (MAPK) pathway is a signaling network that plays important roles in cell proliferation, division, and apoptosis [20]. The three major MAPKs pathways in mammalian cells are the extracellular signal-regulated protein kinase (ERK), p38 kinase, and Jun N-terminal-kinase (JNK) pathways. The phosphatidylinositol 3-kinase (PI3K) pathway affects various cell biological processes, such as proliferation, growth, cell apoptosis, and cell skeletal rearrangement, and AKT, a serine/threonine kinase, plays important roles as a major intracellular signaling pathway [21]. The nuclear factor-κB (NF-κB) family of transcription factors increases important substances related to cell proliferation, apoptosis, and the cell cycle, as well as signal transducers of inflammation and immune responses in several cell types [22]. The NF-κB pathway is regulated by the phosphorylation of its inhibitor, IκB, leading to polyubiquitination and proteosome-mediated degradation. Subsequently, IκB releases the NF-κB dimer, which then translocates to the nucleus and binds with certain DNA-regulating elements, facilitating the expression of downstream target genes. NF-κB activity is also involved in the proliferation and apoptosis of the epithelium [23].

The mechanism of apoptosis can be occasioned by cellular stress, largely classified into extrinsic and intrinsic apoptotic pathways [24,25]. Caspase-9 activation is an essential initiator protein in the process of the intrinsic apoptotic pathway [25]. During the intrinsic pathway, the active form of caspase-9 mediates the apoptosis event by stimulating cleaved forms of effector caspases, such as caspase-3 and caspase-7 [25]. In addition, the intrinsic pathway of apoptosis progressed by cellular damages is involved in the regulation of several cascade molecules including the ratio of the Bcl-2 family members, decreased expression of X-linked inhibitor of apoptosis protein (XIAP), and activation of poly (ADP-ribose) polymerase-1 (PARP-1) [24,25].

Although resveratrol has been shown to have anticancer effects in prostate cancer cells, the mechanisms of action through which resveratrol inhibits the abnormal proliferation of prostate stromal cells have not been fully elucidated. Accordingly, the purpose of this study was to determine the inhibitory effects of resveratrol on cell proliferation in BPH.

## 2. Results

### 2.1. Effects of Resveratrol on Inhibition of WPMY-1 Cell Proliferation

**Figure 1:**
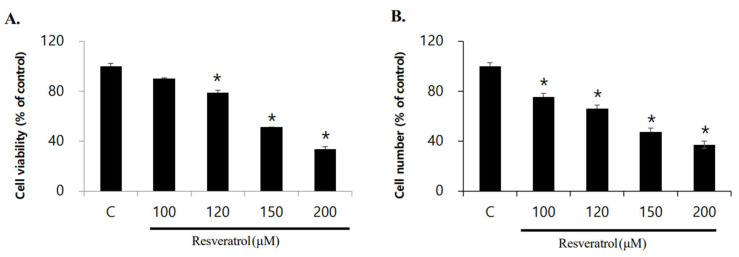
Inhibition of WPMY-1 cell proliferation by resveratrol treatment. The cells were treated with various resveratrol concentrations (0, 100, 120, 150, and 200 μM) for 24 h. (**A**) Cell viability was determined by MTT assay. (**B**) The cells were counted by hemocytometer and microscope. The data are expressed as mean ± SD as a result of three independent experiments depending on the resveratrol concentration (* *p* < 0.05).

**Figure 2:**
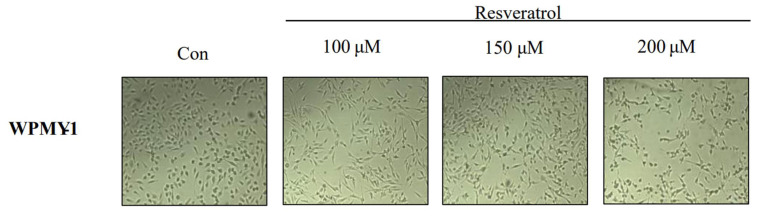
Cellular morphology in WPMY-1 cells treated with resveratrol. The cells were treated without or with various resveratrol concentrations (0, 100, 150, and 200 μM) for 24 h. The morphological change of WPMY-1 cells was imaged using an inverted microscope (original magnification x200).

The effect of resveratrol on the viability of WPMY-1 human prostatic stromal myofibroblast cells was examined. Cells were treated with resveratrol in different concentrations (0, 100, 120, 150, and 200 μM) for 24 h. The number of cells were counted using a microscope, and cell viability was evaluated by 3-(4,5-dimethylthiazol-2-yl)-2,5-diphenyltetrazolium bromide (MTT) assay. We observed concentration-dependent decreases in the viability and number of cells following resveratrol treatment (Figure 1). Based on the above results, we calculated the half-maximal inhibitory concentration of resveratrol in WPMY-1 cells to be approximately 150 μM. In addition, we found that resveratrol induced morphological changes in a concentration-dependent manner (Figure 2).

### 2.2. Effects of Resveratrol on G_0_/G_1_-Phase Cell Cycle Arrest in WPMY-1 Cells

**Figure 3:**
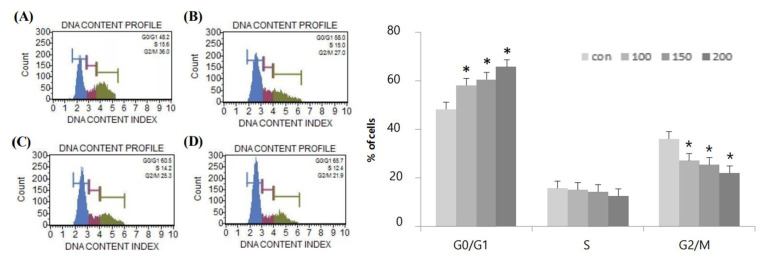
G_0_/G_1_-phase cell cycle arrest of WPMY-1 cells by resveratrol treatment. The cells were treated with various resveratrol concentrations (0, 100, 150, and 200 μM) for 24 h. Flow cytometry analysis was performed to analyze the cell cycle distribution in WPMY-1 cells treated with resveratrol. The results for each concentration are as follows: (**A**) 0 μM, (**B**) 100 μM, (**C**) 150 μM and (**D**) 200 μM. The data are expressed as mean ± SD as a result of three independent experiments depending on the resveratrol concentration (* *p* < 0.05).

Flow cytometry was used to assess the effects of resveratrol on cell cycle arrest in WPMY-1 human prostate stromal myofibroblasts (Table 1). Notably, treatment of WPMY-1 cells with resveratrol (0, 100, 150, or 200 μM) for 24 h induced the accumulation of cells in G_0_/G_1_ phase (48.2%, 58.0%, 60.5%, and 65.7%, respectively). In contrast, we observed decreases in the percentages of cells in S phase (15.6%, 15.0%, 14.2%, and 12.4%, respectively) and G_2_/M phase (36.0%, 27.0%, 25.3%, and 21.9%, respectively). Based on these results, resveratrol was considered to induce G_0_/G_1_ phase arrest in WPMY-1 cells (Table 1 and Figure 3).

### 2.3. Effects of Resveratrol on the Regulation of Cell Cycle-Related Protein Expression in WPMY-1 Cells

**Figure 4:**
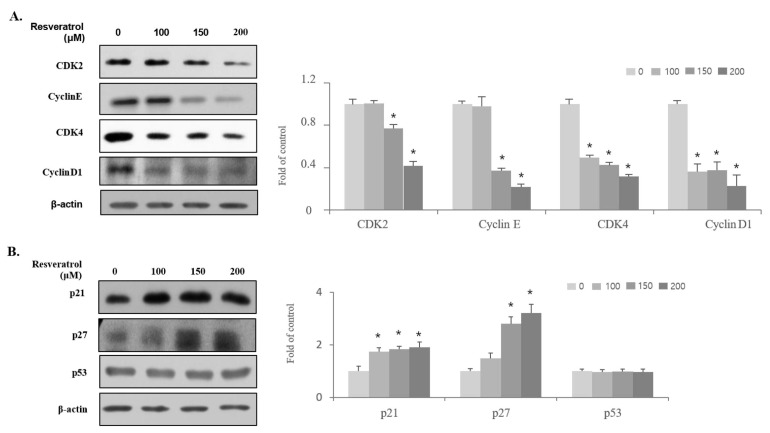
Regulation of cell-cycle-related protein expression in WPMY-1 cells by resveratrol treatment. Cells were incubated with resveratrol for 24 h at the indicated concentrations. (**A**,**B**) Changes in regulatory proteins involved in cell cycle progression were analyzed by immunoblot. β-actin was used as a housekeeping protein in this experiment. The data are expressed as mean ± SD as a result of three independent experiments depending on the resveratrol concentration (* *p* < 0.05).

The mechanisms of cell cycle arrest in G_0_/G_1_ phase induced by resveratrol were investigated. Immunoblot analysis was conducted to examine the expression of G_0_/G_1_-phase regulatory proteins such as CDK2, CDK4, cyclin E, cyclin D1, p21WAF1, p27KIP1, and p53. Figure 4 shows that resveratrol treatment reduced the expression levels of cyclin E, cyclin D1, CDK2, and CDK4 proteins in WPMY-1 cells. In addition, the expression levels of p21_WAF1_ and p27_KIP1_, which are negative regulators of G_0_/G_1_-phase progression and block CDKs, were found to increase, whereas resveratrol treatment did not show any effect on the expression level of the tumor-suppressor p53. These results indicate that resveratrol treatment inhibited the proliferation of WPMY-1 cells through G_0_/G_1_-phase arrest by inducing the expression of p21WAF1 and p27KIP1 along with suppressing the expression of the cyclin E, cyclin D1, CDK2, and CDK4 proteins.

### 2.4. Effects of Resveratrol on Regulation of BPH Markers in WPMY-1 Cells

**Figure 5:**
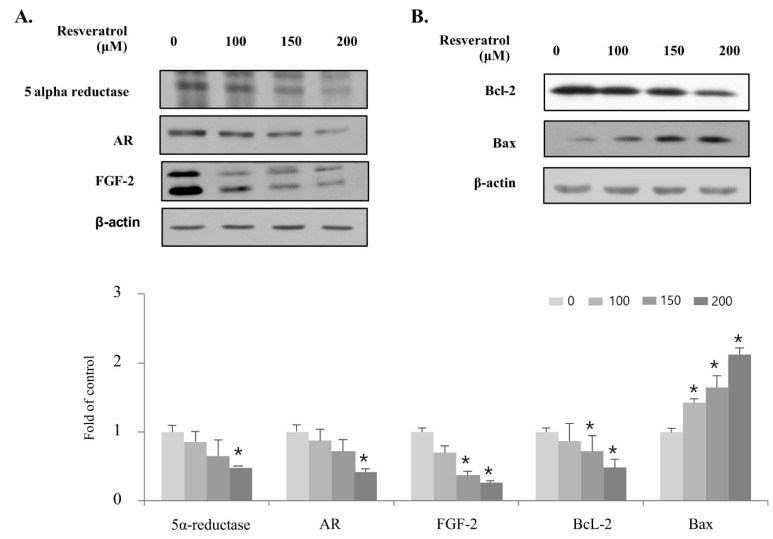
Regulation of the BPH marker expression in WPMY-1 cells by resveratrol treatment. Cells were incubated with resveratrol for 24 h at the indicated concentrations. (**A**,**B**) Changes in the BPH markers involved in prostate development progression were analyzed by immunoblot. β-actin was used as a housekeeping protein in this experiment. The data are expressed as mean ± SD as a result of three independent experiments depending on the resveratrol concentration (* *p* < 0.05).

Next, we investigated whether resveratrol regulated molecular markers associated with proliferation in WPMY-1 cells using immunoblot analysis. Figure 5 shows that treatment with resveratrol induced a decrease in the expression of 5α-reductase, AR, and FGF-2. In addition, the expression levels of Bcl-2, an anti-apoptotic protein that inhibits cell death, were decreased by resveratrol treatment, whereas the expression level of the pro-apoptotic protein Bax was increased. These results indicate that treatment of resveratrol inhibits the proliferation of WPMY-1 cells by inducing the expression of Bax along with suppressing the expression of 5α-reductase, AR, FGF-2, and Bcl-2.

### 2.5. Effects of Resveratrol on the Phosphorylation of the MAPKs and PI3K/AKT Signaling Pathway Components in WPMY-1 Cells

**Figure 6:**
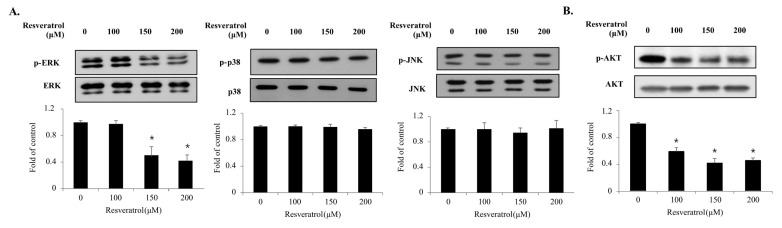
Phosphorylation of the MAPKs and PI3K/AKT signaling in WPMY-1 cells by resveratrol treatment. WPMY-1 cells were treated with various resveratrol concentrations (0, 100, 150, and 200 μM) for 10 min. (**A**) The phosphorylation level of MAPKs was measured by immunoblots. Phosphorylation and total forms of JNK, ERK, and p38 were detected using specific antibodies. (**B**) AKT phosphorylation level was measured by immunoblots. Phosphorylation and total form of AKT were detected using specific antibodies of corresponding AKT. The data are expressed as mean ± SD as a result of three independent experiments depending on the resveratrol concentration (* *p* < 0.05).

The MAPK and PI3K/AKT signaling pathways play important roles in cell proliferation. To elucidate the underlying mechanisms of resveratrol’s action in WPMY-1 cells, we investigated the phosphorylation levels of MAPKs and AKT via immunoblot analysis (Figure 6). Treatment of WPMY-1 cells with resveratrol for 10 min blocked the phosphorylation of ERK1/2 and AKT but did not affect that of JNK1/2 and p38. The results indicate that resveratrol inhibits the proliferation of WPMY-1 cells by suppressing the phosphorylation of the ERK1/2 and AKT signaling pathways.

### 2.6. Effects of Resveratrol on Inhibition of NF-κB Binding Activity in WPMY-1 Cells

**Figure 7:**
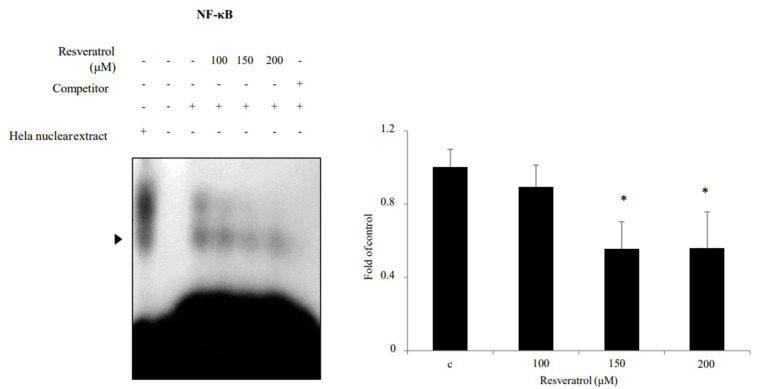
Inhibition of NF-κB binding activity in WPMY-1 cells by resveratrol treatment. WPMY-1 cells were treated with various resveratrol concentrations (0, 100, 150, and 200 μM). Nuclear extracts were collected from the WPMY-1 cells and the binding activity of the NFκB was measured using the EMSA analysis. The data are expressed as mean ± SD as a result of three independent experiments depending on the resveratrol concentration (* *p* < 0.05).

NF-κB activation was identified through electrophoretic mobility shift assay (EMSA). Nuclear extracts from WPMY-1 cells showed reduced NF-κB binding activity when they were treated with resveratrol (Figure 7). The results indicate that resveratrol suppressed NF-κB binding activity in WPMY-1 cells.

### 2.7. Resveratrol Stimulates Apoptosis through Regulating Intrinsic Pathway in WPMY-1 Cells

**Figure 8:**
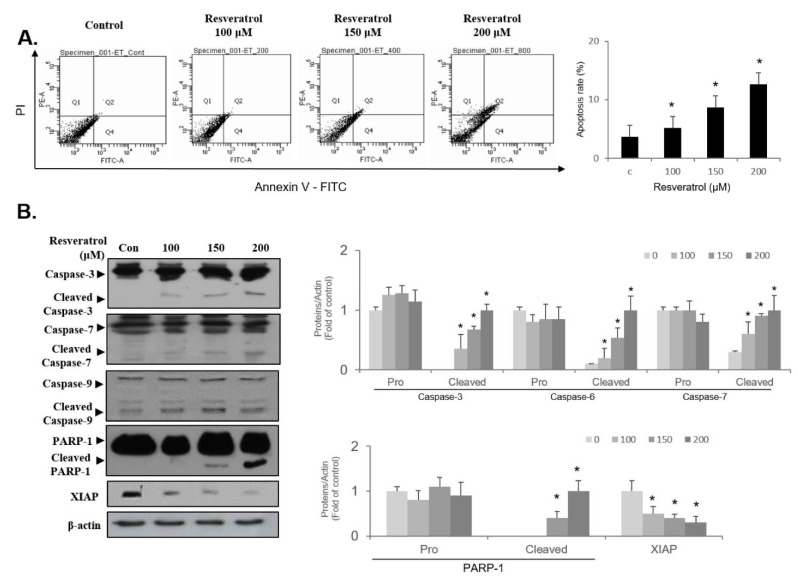
Apoptosis induction in resveratrol-treated WPMY-1 cells. Cells were incubated with various concentrations of resveratrol for 24 h. (**A**) FACS analyses were performed to examine the apoptotic cell death using propidium iodide and annexin V staining. The percentage of apoptotic cells were determined by bar graph compared to controls. (**B**) Changes levels of apoptosis-related regulators in resveratrol-treated WPMY-1 cells were analyzed by immunoblotting. Loading control was presented as β-actin. Bar graphs are given as relative fold changes of each protein in comparison to the control group. Values are expressed as the mean ± SD of three independent experiments; * *p* < 0.05 compared with the control group.

To investigate whether resveratrol triggers apoptosis induction in WPMY-1 cells, FACS analysis was performed using PI and FITC staining. Treatment of cells with resveratrol caused an upregulated level of cells at the late apoptotic phase (Q2) at 24 h in a concentration-dependent fashion (Figure 8A). Since resveratrol treatment increased the level of Bax and decreased the level of Bcl-2 (Figure 5B), we next examined the expression level of apoptosis proteins associated with intrinsic pathway in resveratrol-treated cells. As shown in Figure 8B, resveratrol treatment activated intrinsic apoptosis signaling molecules such as caspase-9, caspase-3, and caspase-7 in WPMY-1 cells. In addition, activation of PARP-1 was observed in resveratrol-treated cells (Figure 8B). Furthermore, the expression levels of XIAP proteins were decreased following treatment with resveratrol (Figure 8B). These results demonstrate that resveratrol induces the intrinsic-related apoptosis pathway in WPMY-1 cells.

## 3. Discussion

The physiologically active functions of resveratrol, including its antioxidant, cytoprotective, anti-migratory, and cell growth inhibitory effects, may contribute to the suppression of prostatic hyperplasia [15,26,27]. We aimed to investigate the effects of resveratrol in WPMY-1 prostate stromal cells. Our findings showed that resveratrol blocked cell proliferation by inducing G_1_-phase arrest in WPMY-1 cells. Moreover, resveratrol suppressed CDK2, cyclin E, CDK4, and cyclin D1 expression and promoted p21_WAF1_ and p27_KIP1_ expression. These findings provide insight into the mechanisms of resveratrol in WPMY-1 cells.

Prostatic hypertrophy is regulated by the expression levels of molecular markers, such as 5α-reductase, AR, FGF-2, Bcl-2, and Bax [15]. In this study, treatment of WPMY-1 prostate stromal cells with resveratrol downregulated 5α-reductase, AR, FGF-2, and Bcl-2, but upregulated Bax. Similar to the results of a previous study, these findings show that resveratrol suppresses the proliferation of prostate stromal cells by controlling the levels of molecular markers relevant to growth and cell death, thereby affecting BPH development and progression [15].

The PI3K/AKT and MAPK signaling pathways are important in cellular activities, including cell growth and proliferation [20,21]. It has been reported that the phosphorylation of AKT and ERK is essential for the regulation of prostate diseases [28,29,30]. In this study, resveratrol suppressed the phosphorylation of ERK1/2 and AKT but did not show any effect on the phosphorylation of p38 and JNK, similar to the findings of previous studies. Thus, these findings suggest that resveratrol blocks prostate cell proliferation by inhibiting the ERK and AKT signaling pathways. In addition, resveratrol downregulated NF-κB levels in prostate cells. In most cells, the activity of NF-κB generates pro-survival signals, including cell differentiation, cell proliferation, and cell death [23]. Hence, a decrease in NF-κB transcriptional activity by resveratrol may induce apoptosis. Additionally, resveratrol modulates the levels of NF-κB-mediated microRNAs [31]. Previous studies have reported that the activity of NF-κB promotes the continuous transcription of proliferative genes by maintaining the activity of the AR, which has central roles in the progression and development of prostate disease [28]. Taken together, these findings highlight the function of NF-κB as an important regulator of resveratrol-dependent inhibition of human prostate cell proliferation.

Apoptosis is a crucial program in the control of the cell death mechanism [24,25]. It comprises well-known signaling pathways involving independent effector caspases, including an intrinsic pathway (Bcl-2-related cascade) and an extrinsic pathway (Fas-related cascade) [24,25]. FACS analysis with PI and FITC staining showed the accumulation of late apoptotic cell phase in resveratrol-treated WPMY-1 cells, indicating that the resveratrol-induced suppression of cell proliferation is closely associated with apoptosis pathways. Immunoblot results revealed the reduction of Bcl-2 and the induction of Bax. These results led us to investigate the intrinsic apoptosis pathway. Many studies have addressed whether cellular damage or stress could upregulate the Bax/Bcl-2 ratio, which in turn would activate the initiator molecule caspase-9 during the intrinsic apoptosis pathway [25]. Subsequently, the activation of caspase-9 was described as triggering the downstream effectors caspase-3 and caspase-7 [25]. Caspase-3 activation stimulates apoptosis signaling via the induction of the cleavage of PARP-1, resulting in the limitation of the DNA repair system in eukaryotic cells [29,30]. Previous studies have shown that XIAP, an anti-apoptotic molecule, is involved in the prevention of apoptosis by binding to members of the caspase family, such as caspase-9, caspase-3, and caspase-7 [24,25]. In the present study, resveratrol upregulated the ratio of Bax/Bcl-2 in WPMY-1 cells, which resulted in the activation of caspase-9, and subsequently led to the induction of the cleaved forms of both caspase-3 and caspase-7. Treatment with resveratrol stimulated the activation of PARP-1 via the upregulation of cleaved forms of PARP-1. In addition, the expression level of the anti-apoptotic molecule XIAP was decreased in resveratrol-treated cells. Our results were supported by the resveratrol-induced intrinsic apoptosis pathway, involving the occurrence of Bcl-2 family/caspase-9/XIAP/caspase-3 or capsase-7/PARP-1 cascade in WPMY-1 cells.

In conclusion, we found that resveratrol inhibited cell proliferation by inducing G_1_-phase arrest via the regulation of cyclin E, cyclin D1, CDK2, CDK4, p21_WAF1_, and p27_KIP1_ expression in WPMY-1 cells. In addition, resveratrol inhibited cell proliferation by modulating the expression levels of BPH-related molecules, including 5α-reductase, FGF-2, Bcl-2, and Bax. Treatment with resveratrol also suppressed the AKT and ERK1/2 signaling pathways and inhibited NF-κB binding activity. Furthermore, resveratrol treatment promoted apoptosis via the regulation of the intrinsic pathway. These findings provide important insights into the molecular mechanisms through which resveratrol exerts antiproliferative effects in WPMY-1 prostate stromal cells, establishing resveratrol as a potential therapeutic agent in the prevention or treatment of BPH.

## 4. Materials and Methods

### 4.1. Chemicals

Resveratrol (≥98%, analytical standard grade) was purchased from Sigma–Aldrich (St. Louis, MO, USA). Polyclonal antibodies against extracellular signal-regulated kinase ERK (9102S), phospho-ERK (9101S), p38 mitogen-activated protein kinase (9212S), phospho-p38 MAPK (9211S), Jun N-terminal-kinase (9258S), phospho-JNK (9251S), AKT (9272S), phospho-AKT (9272S), and p21WAF1 (2947S) were obtained from Cell Signaling Technology Inc. (Danvers, MA, USA). Polyclonal antibodies against CDK2 (sc-163), cyclin E (sc-377100), CDK4 (sc-23896), cyclin D1 (sc-8396), p53 (sc-1641), p27KIP1 (sc-1641), β-actin (sc-47778), 5α-reductase 2 (sc-293232), AR (sc-7305), FGF-2 (sc-1360), B-cell lymphoma-2 (sc-7382) and Bcl-2-associated x protein (sc-20067) were obtained from Santa Cruz Biotechnology Inc. (Santa Cruz, CA, USA). A nuclear extract kit and electrophoretic mobility shift assay (EMSA) gel shift kit were obtained from Panomics (Fremont, CA, USA). Polyclonal antibodies against PARP-1 (sc-7150), caspase-9 (sc-7885), caspase-7 (9492S), caspase-3 (sc-7148), cleaved PARP-1 (sc-7150), cleaved caspase-9 (sc-7885), cleaved caspase-7 (9492S), cleaved caspase-3 (sc-7148), and XIAP (sc-11426) were obtained from Santa Cruz Biotechnology (Santa Cruz, CA, USA).

### 4.2. Cell Cultures

Human normal prostate stromal cells (WPMY-1) were purchased from American Type Culture Collection (ATCC, Baltimore, MD, USA). WPMY-1 cells were maintained in Dulbecco Modified Eagle Medium (DMEM). The medium was supplemented with 1% penicillin-streptomycin (Gibco) and 5% fetal bovine serum (FBS). Cells were incubated in an incubator at 37 °C with an atmosphere of 5% CO_2_ in air.

### 4.3. Cell Viability Assay

MTT assay was performed to assess cell viability. Briefly, 96-well plates seeded with 3 × 10^3^ cells were incubated overnight in a CO_2_ incubator set at 37 °C. Resveratrol diluted in dimethyl sulfoxide (DMSO) was treated with various concentrations on the seeded cells for 24 h. Subsequently, the cells were incubated for another 4 h after the addition of 10 μL of MTT solution (0.5 mg/mL). After removing the supernatants from the wells of the plate, the cells were dissolved in 100 μL of added DMSO. The absorbance was measured at 570 nm using a microplate reader.

### 4.4. Cell Counting

After seeding, cells were treated with different concentrations of resveratrol for 24 h and separated from the plate by trypsinization. Trypsin-treated cells were gently pipetted to blend with 0.4% Trypan Blue (Sigma–Aldrich, St. Louis, MO, USA) and then counted immediately by a hematocytometer.

### 4.5. Flow Cytometric Analysis

WPMY-1 cells were trypsinized, fixed with ethanol (70%), washed with cold phosphate-buffered saline (PBS), and incubated with RNase and propidium iodide (Sigma-Aldrich). Flow cytometry (FACStar; BD Biosciences, San Jose, CA, USA) equipped with BD Cell Fit software was used to measure the cell cycle distribution.

### 4.6. Immunoblotting

After washing with cold PBS, cells were gently lysed in lysis buffer (250 µL containing HEPES (50 mM, pH 8.0), NaCl (150 mM), ethylenediaminetetraacetic acid (EDTA, 1 mM), ethylene glycol tetra acetic acid (EGTA, 2.5 mM), phenylmethylsulfonyl fluoride (PMSF, 0.1 mM), dithiothreitol (DTT, 1 mM), Na_3_VO_4_ (0.1 mM), 10% glycerol, 0.1% Tween-20, leupeptin (10 g/mL), β-glycerophosphate (10 mM), and aprotinin (2 µg/mL)). Then the cells were collected in microtubes and kept on ice for 10 min before centrifugation (13,000 rpm for 15 min at 4 °C). Protein concentrations in cells were analyzed by a BCA reagent kit (Thermo Scientific, Rockford, IL, USA). An amount of 20 μg cellular protein was resolved by sodium dodecyl sulfate polyacrylamide gel electrophoresis on 7.5%, 10%, and 12% polyacrylamide gels. The gel with protein was transferred into a nitrocellulose membrane (Hybond, Amersham Corp) using electrophoresis. The nitrocellulose membranes were blocked in 5% BSA (MP Biomedicals, OH, USA) and 5% skim milk for 2 h, and incubated with primary antibodies (1:1000 dilution) at 4 °C for 24 h, and secondary antibodies (1:5000 dilution) for 2 h. Subsequently, immunocomplexes were analyzed using enhanced chemiluminescence (ECL) immunoblotting detection reagents (Supersignal, Thermo Scientific, Rockford, IL, USA).

### 4.7. EMSA (Nuclear Extracts and Electrophoretic Mobility Shift Assay)

Collected cells in lysis buffer (10 mM HEPES (pH 8.0], EDTA (0.1 mM], KCl (10 mM), EGTA (0.1 mM), PMSF (0.5 mM), and DTT (1 mM)) were vortexed in 0.5% Nonidet P-40 and centrifuged at 13,000 rpm (4 °C, 15 min). The cellular pellets were resuspended for 15 min in a cold high-salt buffer (20 mM HEPES (pH 8.0), EDTA (1 mM), EGTA (1 mM), NaCl (0.4 M], PMSF (1 mM), and DTT (1 mM)). The amount of nuclear protein was detected by a BCA reagent kit. The probe for the consensus oligonucleotide sequences of NF-κB was AGTTGAGGGGACTTTCCCAGGC, and EMSA was performed by annealing oligonucleotides in an annealing buffer (EDTA (1 mM), Tris (10 mM, pH 8) and NaCl (50 mM)) with heating for 2 min at 90 °C. NF-κB oligonucleotides were finally labeled by 1 h incubation of T4 polynucleotide kinase (Promega, WI, USA) with [32P] APT at 37 °C. Nuclear extract (5 μg) extracted from cells was incubated in 2× binding buffer (HEPES (25 mM, pH 8.0), EDTA (1 mM), DTT (0.5 mM), MgCl_2_ (5 mM), KCl (75 mM), glycerol (10%), and poly dI/dC (0.25 μg/mL)) for 20 min at 25 °C with ^32^P-labeled oligonucleotide probe. Electrophoresis was conducted to detect the DNA–protein complex on a 6% polyacrylamide gel using 0.5× TBE running buffer. The gel was washed, dried, and then kept overnight at −70 °C to visualize via automatic radiography using X-ray film.

### 4.8. Apoptosis Analysis by Flow Cytometry

Apoptosis assay was performed using a BioVision Annexin V-FITC apoptosis detection kit (BioVision Inc., Milpitas, CA, USA). Briefly, cell suspensions were reacted with PI (5 μL) and FITC-Annexin V (5 μL) at room temperature in the dark. After 15 min incubation, the cells were analyzed via the use of flow cytometry (FACSCalibur; Becton-Dickinson, San Jose, CA, USA). The percentage of cells in different stages were evaluated as follows: living cells (Q1; Annexin V−/PI−), early apoptotic/primary apoptotic cells (Q4; Annexin V+/PI−), late apoptotic/secondary apoptotic cells (Q2; Annexin V+/PI+), and necrotic cells (Q4; Annexin V−/PI+). All analyses were performed by three independent experiments.

### 4.9. Statistical Analysis

All data are shown as mean ± standard error (SE) of 3 repeated experiments. Statistical analyses were conducted by SPSS version 25 (SPSS Inc., Chicago, IL, USA) and Duncan’s multiple range test was used as a post-test to confirm the differences between pairs of groups. A value of *p* < 0.05 was used to represent a statistically significant difference.

## Figures and Tables

**Table 1:** Percentages of cells in G_0_/G_1_, S, and G_2_/M phases.

Resveratrol Concentration (μM)	G_0_/G_1_	S	G_2_/M
0	48.2%	15.6%	36.0%
100	58.0%	15.0%	27.0%
150	60.5%	14.2%	25.3%
200	65.7%	12.4%	21.9%

## Data Availability

The data presented in this study are available on request from the corresponding author.

## Abbreviations

AKT	Alpha serine/threonine-protein kinase
CDK	Cyclin-dependent kinase
EMSA	Electrophoretic mobility shift assay
ERK	Extracellular signal-regulated kinase
JNK	c-Jun N-terminal protein kinase
MAPKs	Mitogen-activated protein kinases
mRNA	Messenger ribonucleic acid
NF-κB	Nuclear factor kappa-light-chain-enhancer of activated B cells
P38 MAPK	p38 mitogen-activated protein kinase
PI3K/AKT	Phosphoinositide 3-kinase/AKT
XIAP	X-linked inhibitor of apoptosis protein
PARP-1	Poly (ADP-ribose) polymerase-1

## References

[1] Khusbu F.Y., Zhou X., Roy M., Chen F., Cao Q., Chen H. (2020). Resveratrol Induces Depletion of TRAF6 and Suppresses Prostate Cancer Cell Proliferation and Migration. Int. J. Biochem. Cell Biol..

[2] Sobolev V.S., Cole R.J. (1999). Trans-Resveratrol Content in Commercial Peanuts and Peanut Products. J. Agric. Food Chem..

[3] Pavan A.R., Silva G.D.B.D., Jornada D.H., Chiba D.E., Fernandes G.F.D.S., Man Chin C., Dos Santos J.L. (2016). Unraveling the Anticancer Effect of Curcumin and Resveratrol. Nutrients.

[4] Berry S.J., Coffey D.S., Walsh P.C., Ewing L.L. (1984). The Development of Human Benign Prostatic Hyperplasia with Age. J. Urol..

[5] Ilic D., Misso M. (2012). Lycopene for the Prevention and Treatment of Benign Prostatic Hyperplasia and Prostate Cancer: A Systematic Review. Maturitas.

[6] Park E., Lee M., Seo C., Jeon W., Shin H. (2017). Yongdamsagan-Tang, a Traditional Herbal Formula, Inhibits Cell Growth through the Suppression of Proliferation and Inflammation in Benign Prostatic Hyperplasia Epithelial-1 Cells. J. Ethnopharmacol..

[7] Trachtenberg J., Hicks L.L., Walsh P.C. (1980). Androgen-and Estrogen-Receptor Content in Spontaneous and Experimentally Induced Canine Prostatic Hyperplasia. J. Clin. Investig..

[8] Barrack E.R., Berry S.J. (1987). DNA Synthesis in the Canine Prostate: Effects of Androgen and Estrogen Treatment. Prostate.

[9] Diez G. (1998). Zonal Variation of Apoptosis and Proliferation in the Normal Prostate and in Benign Prostatic Hyperplasia. Br. J. Urol..

[10] Tao R., Miao L., Yu X., Orgah J.O., Barnabas O., Chang Y., Liu E., Fan G., Gao X. (2019). Cynomorium Songaricum Rupr Demonstrates Phytoestrogenic Or Phytoandrogenic Like Activities that Attenuates Benign Prostatic Hyperplasia Via Regulating Steroid 5-A-Reductase. J. Ethnopharmacol..

[11] Goldenberg L., So A., Fleshner N., Rendon R., Drachenberg D., Elhilali M. (2009). The Role of 5-Alpha Reductase Inhibitors in Prostate Pathophysiology: Is there an Additional Advantage to Inhibition of Type 1 Isoenzyme?. CUAJ.

[12] Lee K.L., Peehl D.M. (2004). Molecular and Cellular Pathogenesis of Benign Prostatic Hyperplasia. J. Urol..

[13] Kyprianou N., Tu H., Jacobs S.C. (1996). Apoptotic Versus Proliferative Activities in Human Benign Prostatic Hyperplasia. Hum. Pathol..

[14] Wu W., Maneix L., Insunza J., Nalvarte I., Antonson P., Kere J., Yu N.Y., Tohonen V., Katayama S., Einarsdottir E. (2017). Estrogen Receptor Β, a Regulator of Androgen Receptor Signaling in the Mouse Ventral Prostate. Proc. Acad. Natl. Sci. USA.

[15] Song J.H., Hwang B., Chung H.J., Moon B., Kim J.W., Ko K., Kim B.W., Kim W.R., Kim W.J., Myung S.C. (2020). Peanut Sprout Extracts Cultivated with Fermented Sawdust Medium Inhibits Benign Prostatic Hyperplasia in Vitro and in Vivo. World J. Mens. Health..

[16] Zhong X., Lin J., Zhou J., Xu W., Hong Z. (2015). Anti-Proliferative Effects of Qianliening Capsules on Prostatic Hyperplasia in Vitro and in vivo. Mol. Med. Rep..

[17] Hartwell L.H., Weinert T.A. (1989). Checkpoints: Controls that Ensure the Order of Cell Cycle Events. Science.

[18] Xiong Y., Hannon G.J., Zhang H., Casso D., Kobayashi R., Beach D. (1993). P21 is a Universal Inhibitor of Cyclin Kinases. Nature.

[19] Toyoshima H., Hunter T. (1994). p27, a Novel Inhibitor of G1 Cyclin-Cdk Protein Kinase Activity, is Related to p21. Cell.

[20] Deane A.R., Potemkin N., Ward R.D. (2020). Mitogen-Activated Protein Kinase (MAPK) Signalling Corresponds with Distinct Behavioural Profiles in a Rat Model of Maternal Immune Activation. Behav. Brain Res..

[21] Vivanco I., Sawyers C.L. (2002). The Phosphatidylinositol 3-kinase–AKT Pathway in Human Cancer. Nat. Rev. Cancer.

[22] Chae S.W. (2005). Function and Activation of NF-Kappa B in Immune System. Korean J. Otorhinolaryngol. Head Neck Surg..

[23] Brantley D.M., Chen C., Muraoka R.S., Bushdid P.B., Bradberry J.L., Kittrell F., Medina D., Matrisian L.M., Kerr L.D., Yull F.E. (2001). Nuclear Factor-κB (NF-κB) Regulates Proliferation and Branching in Mouse Mammary Epithelium. Mol. Biol. Cell.

[24] Soldani C., Scovassi A.I. (2002). Poly(ADP-Ribose) Polymerase-1 Cleavage during Apoptosis: An Update. Apoptosis.

[25] de Almagro M.C., Vucic D. (2012). The Inhibitor of Apoptosis (IAP) Proteins are Critical Regulators of Signaling Pathways and Targets for Anti-Cancer Therapy. Exp. Oncol..

[26] Fremont L. (2000). Biological Effects of Resveratrol. Life Sci..

[27] Hsieh T.C., Wu J.M. (2020). Resveratrol suppresses prostate cancer epithelial cell scatter/invasion by targeting inhibition of hepatocyte growth factor (HGF) secretion by prostate stromal cells and upregulation of E-cadherin by prostate cancer epithelial cells. Int. J. Mol. Sci..

[28] Lamont K.R., Tindall D.J. (2011). Minireview: Alternative Activation Pathways for the Androgen Receptor in Prostate Cancer. J. Mol. Endocrinol..

[29] Zhang Z., Duan L., Du X., Ma H., Park I., Lee C., Zhang J., Shi J. (2008). The Proliferative Effect of Estradiol on Human Prostate Stromal Cells is Mediated through Activation of ERK. Prostate.

[30] Luo Y., Wu J., Lu M., Shi Z., Na N., Di J. (2016). Carvacrol Alleviates Prostate Cancer Cell Proliferation, Migration, and Invasion through Regulation of PI3K/Akt and MAPK Signaling Pathways. Oxid. Med. Cell. Longev..

[31] Xu L., Botchway B.O., Zhang S., Zhou J., Liu X. (2018). Inhibition of NF-κB Signaling Pathway by Resveratrol Improves Spinal Cord Injury. Front. Neurosci..

